# Impact of allogeneic red blood cell transfusion on prognosis in soft tissue sarcoma patients. A single‐centre study

**DOI:** 10.1002/cam4.4989

**Published:** 2022-06-28

**Authors:** Maria Anna Smolle, Wolfgang Helmberg, Eva Maria Matzhold, Dominik Andreas Barth, Nazanin Sareban, Joanna Szkandera, Bernadette Liegl‐Atzwanger, Andreas Leithner, Martin Pichler

**Affiliations:** ^1^ Department of Orthopaedics and Trauma Medical University of Graz Graz Austria; ^2^ Department of Blood Group Serology and Transfusion Medicine Medical University of Graz Graz Austria; ^3^ Division of Clinical Oncology, Department of Internal Medicine Medical University of Graz Graz Austria; ^4^ Diagnostic and Research Institute of Pathology Medical University of Graz Graz Austria

**Keywords:** prognosis, soft tissue sarcoma, surgery, surgical oncology

## Abstract

**Background:**

Perioperatively administered (leukocyte reduced) allogeneic red blood cell transfusions (lrRBCTs) may lead to transfusion‐related immunomodulation and reduced overall survival (OS) in cancer patients. Herein, the effect of lrRBCT on local recurrence (LR), distant metastasis (DM), and OS in soft tissue sarcoma (STS) patients was analysed.

**Methods:**

Retrospective study on 432 STS patients (mean age: 60.0 ± 17.8 years; 46.1% female), surgically treated at a tertiary tumour centre. Uni‐ and multivariate survival models were calculated to analyse impact of perioperative lrRBCTs on LR, DM, OS.

**Results:**

Perioperatively, 75 patients (17.4%) had received lrRBCTs. Older patients, deep, large, lower limb STS rather required lrRBCTs (all *p* < 0.05). No significant association between lrRBCT administration and LR‐ (*p* = 0.582) or DM‐risk (*p* = 0.084) was observed. LrRBCT was associated with worse OS in univariate analysis (HR: 2.222; *p* < 0.001), with statistical significance lost upon multivariate analysis (HR: 1.658; *p* = 0.059; including age, histology, size, grading, amputation, depth). Adding preoperative haemoglobin in subgroup of 220 patients with laboratory parameters revealed significant negative impact of low haemoglobin on OS (*p* = 0.014), whilst effect of lrRBCT was further diminished (*p* = 0.167).

**Conclusion:**

Unfavourable prognostic factors prevail in STS patients requiring lrRBCTs. Low haemoglobin levels rather than lrRBCT seem to reduce OS.

## Graphical abstract

Unfavourable prognostic factors prevail in patients with soft tissue sarcoma requiring red blood cell transfusions upon surgery. Survival in soft tissue sarcoma patients is rather associated with low haemoglobin levels than necessity for red blood cell transfusion.

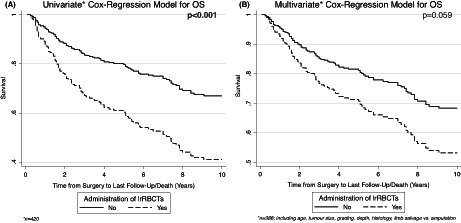

## 1 INTRODUCTION

Perioperative leukocyte reduced red blood cell transfusions (lrRBCTs) can be life‐prolonging and ‐saving in cancer patients with anaemia, either caused by the tumour's metabolism, cytotoxic chemotherapy, or excessive blood loss during surgery. Whilst slowly developing anaemia may be approached by erythropoesis stimulating agents (ESA) and/or iron substitution, abrupt drops in haemoglobin levels are usually treated with leukocyte reduced red blood cell (lrRBC) units in case the patient becomes symptomatic or transfusions triggers have been exceeded.^1^ The prevalence of anaemia in cancer patients is nearly 40%.^2^ In cancer patients in general,^2^, ^3^, ^4^ and in STS patients,^5^ anaemia is not only significantly associated with an impaired quality of life,^2^, ^3^, ^4^ but also reduced survival.^2^, ^3^, ^4^, ^5^

However, administration of allogeneic lrRBC units has come under criticism as it may lead to transfusion‐related immunomodulation, a complex mechanism involving enhanced release of immunosuppressive prostaglandins, alteration of T‐cell and monocyte activity, and diminished production of interleukin‐2.^1^, ^6^, ^7^ Both leukocytes prevailing in lrRBCTs after leukoreduction as well as associated by‐products may contribute to these processes, eventually promoting tumour progression, and reducing overall survival (OS).^1^, ^6^, ^8^

In soft tissue sarcoma (STS), a rare neoplasm of mesenchymal origin, the administration of lrRBC units has been associated with increased risk for distant metastasis (DM),^9^ and reduced OS.^9^, ^10^ Notably, results on effects of lrRBCT were based on studies involving 200 patients or less, treated in 2006 or prior.^9^, ^10^ As safety of allogeneic lrRBCT has significantly improved over the years based on refined methods to reduce pathogen load and enhance donor‐recipient compatibility,^11^ the current study evaluated the potential prognostic role of lrRBCT in STS patients treated from 1998 to 2016 at a single tertiary tumour centre.

The aims of this study were to analyse (1) the frequency of perioperative allogeneic lrRBCT in STS patients consecutively treated at a tertiary sarcoma centre, (2) factors associated with administration of lrRBC units, (3) and the impact of lrRBCT on local recurrence (LR) and DM, as well OS.

## 2 METHODS

Altogether, 432 Caucasian STS patients who were consecutively treated between 1998 and 2016 at a single tertiary sarcoma centre were retrospectively included. All patients had undergone surgery for primary localised disease with curative intention. Mean patient age was 60.0 ± 17.8 years, and 199 patients were female (46.1%). The study was approved by the local ethics committee (IRB‐number: 32–225 ex 19/20).

Demographic, tumour‐ and treatment‐related variables were ascertained from medical records as well as radiological, surgical and histopathological reports. Histological subtypes were subdivided into 5 categories: myxofibrosarcoma, synovial sarcoma, undifferentiated pleomorphic sarcoma, liposarcoma, and others. Depth was divided into three categories depending on the location of the tumour relative to the fascia, i.e. superficial (above fascia), deep (below fascia), as well as superficial and deep combined (tumour breaching the fascia). For tumour size, the largest diameter of the tumour, either based on definite resection specimen or preoperative imaging, was used. The FNLCC (*Fédération Française des Centres de Lutte Contre le Cancer*) grading system was applied to categorise STS into low (=G1), intermediate (=G2), or high‐grade (=G3).^12^ Liposarcomas graded G1 were excluded as nowadays they are regarded as non‐metastasising (atypical lipomatous tumour).^13^ Margins were defined as negative (=R0, microscopically negative), and marginal/intralesional (=R1/2, microscopically/macroscopically positive).

As only 8 and 11 patients of the entire dataset had received neoadjuvant radiotherapy (RTX) and chemotherapy (CTX), respectively, timing of RTX was omitted and patients classified either as having received RTX and CTX at any time point or not.

Information on allogeneic lrRBCT (containing per definition <1 × 10^6^ white‐blood cells per unit) was taken from the *Department of Blood Group Serology and Transfusion Medicine* affiliated to the same university as the tertiary sarcoma centre. The perioperative period was defined as lrRBC units transfused 7 days prior to 14 days after definite surgery. LrRBC units and exact volume (ml) transfused, as well as median age of lrRBC units per patient at time of transfusion were ascertained. In case only one lrRBC unit had been administered perioperatively, this unit's age was used. For statistical analyses, median age of lrRBC units per patient was split at the median age of the entire cohort.

Notably, laboratory parameters (leukocytes [in g/L], erythrocytes [in 10^12^/L], haemoglobin [in g/L], C‐reactive protein [CRP] levels [in g/dl]) deriving from pre‐surgical blood tests could be obtained from 220 patients of the entire cohort (50.9%). From the same group of patients, postoperative laboratory parameters from day 1 to 3 following surgery (or after first postoperative lrRBCT) could be ascertained in 211 individuals.

Patients were followed‐up regularly adhering to follow‐up regimens in its respective current version. Date of LR and DM was defined as first image‐based diagnosis. Date of last follow‐up or death was defined as the last patient contact, either based on entries in medical records, or most recent telephone calls performed by study nurses. OS was calculated from definite surgery to last follow‐up or death. Median follow‐up of all patients was 46.0 months (IQR: 19.5–96.0 months). The study was performed according to the STROBE statement.^14^

### 2.1 Statistical analysis

For normally and non‐normally distributed variables, means and medians were provided with corresponding standard deviations and interquartile ranges (IQR), respectively. T‐tests and chi‐squared tests were performed to assess differences between continuous and binary, or two binary/categorical variables. To assess changes in laboratory parameters from pre‐ to postoperative depending on administration of lrRBCTs, calculated differences in haemoglobin and CRP levels were compared with t‐tests. Impact of prognostic variables on LR‐ and DM‐risk were assessed with univariate and multivariate Fine&Grey models, with death as competing event. LR‐models were calculated after excluding patients having undergone amputation (*n* = 36). Univariate and multivariate Cox‐regression models were used to analyse the impact of prognostic variables on OS. Parameters significantly associated with the outcome in the univariate analyses were included in the respective multivariate models, together with administration of lrRBCTs. Subhazard ratios (SHRs), hazard ratios (HRs) and corresponding 95% confidence intervals (CIs) were provided for the respective time‐to‐event models.

A *p*‐value of <0.05 was considered statistically significant.

## 3 RESULTS

With 63.1%, the majority of STS were located in the lower limbs (*n* = 272). Moreover, most STS were situated deep to the fascia (*n* = 236; 54.9%). Mean tumour size was 7.8 ± 5.3 cm. The most common histological subtype was myxofibrosarcoma in 34.7% of cases (*n* = 150), followed by undifferentiated pleiomorphic sarcoma in 14.8% (*n* = 64), and liposarcoma in 12.7% (*n* = 55). Of the entire cohort, 36 patients underwent amputation (8.4%). Twenty‐nine (6.7%), 163 (37.8%), and 36 (8.4%) patients required (neuro‐)vascular, plastic, and endoprosthetic reconstruction during definite surgery, respectively. Further demographic, tumour‐ and treatment‐related variables, separated by lrRBCT administration, are listed in Table 1.

**TABLE 1:** Descriptive analysis, split by administration of lrRBCT

		Missing	Overall	lrRBCT		*p*‐value
				No (*n* = 357)	Yes (*n* = 75)	
Age (mean ± standard deviation)		0	59.9 ± 17.8	58.9 ± 17.6	64.8 ± 18.2	**0.008**
Gender	*Male*	0	233 (53.9)	197 (84.6)	36 (15.4)	0.257
	*Female*		199 (46.1)	160 (80.4)	39 (19.6)	
Location	*Upper limb*	1	116 (26.9)	104 (89.7)	12 (10.3)	**0.050**
	*Lower limb*		272 (63.1)	216 (79.4)	56 (20.6)	
	*Trunk*		43 (10.0)	36 (83.7)	7 (16.3)	
Tumour size (mean ± standard deviation)		9	7.8 ± 5.3	6.6 ± 4.2	13.2 ± 6.3	**<0.001**
Grading	*G1*	28	39 (9.7)	39 (100.0)	0 (0.0)	**0.004**
	*G2*		97 (24.0)	81 (83.5)	16 (16.5)	
	*G3*		268 (66.3)	210 (78.4)	58 (21.6)	
Depth	*Superficial*	2	137 (31.9)	128 (93.4)	9 (6.6)	**<0.001**
	*Deep*		236 (54.8)	181 (76.7)	55 (23.3)	
	*Superficial + Deep*		57 (13.3)	46 (80.7)	11 (19.3)	
Histology	*Myxofibrosarcoma*	0	150 (34.7)	128 (85.3)	22 (14.7)	**0.022**
	*Synovial sarcoma*		32 (7.4)	29 (90.6)	3 (9.4)	
	*UPS*		64 (14.8)	54 (84.4)	10 (15.6)	
	*Liposarcoma*		55 (12.7)	37 (67.3)	18 (32.7)	
	*Other*		131 (30.4)	109 (83.2)	22 (16.8)	
Margins	*R0*	1	349 (81.0)	294 (84.2)	55 (15.8)	0.064
	*R1/2*		82 (19.0)	62 (75.6)	20 (24.4)	
Amputation	*No*	1	395 (91.6)	328 (83.0)	67 (17.0)	0.425
	*Yes*		36 (8.4)	28 (77.8)	8 (22.2)	
(Neuro)‐vascular Reconstruction	*No*	1	402 (93.3)	337 (83.8)	65 (16.2)	**0.012**
	*Yes*		29 (6.7)	19 (65.5)	10 (34.5)	
Plastic Reconstruction	*No*	1	268 (62.2)	220 (82.1)	48 (17.9)	0.721
	*Yes*		163 (37.8)	136 (83.4)	27 (16.6)	
Endoprosthetic Reconstruction	*No*	1	395 (91.6)	335 (84.8)	60 (15.2)	**<0.001**
	*Yes*		36 (8.4)	21 (58.3)	15 (41.7)	
Any CTX	*No*	1	378 (87.7)	317 (83.9)	61 (16.1)	0.065
	*Yes*		53 (12.3)	39 (73.6)	14 (26.4)	
Any RTX	*No*	28	149 (36.9)	118 (79.2)	31 (20.8)	0.192
	*Yes*		255 (63.1)	215 (84.3)	40 (15.7)	

*Note*: *p*‐values calculated with chi‐squared tests for binary/categorical variables or *t*‐tests for continuous variables.

*p*‐values in bold highlight significant results.

Overall, only 75 patients received allogeneic lrRBC units perioperatively (17.4%). Of these, two (2.7%) had received lrRBCTs preoperatively (day 2 and 5), whilst 53 underwent transfusion at day of surgery (70.7%), and 20 in the postoperative period (26.6%). Notably, information on exact amount of lrRBCTs given to patients was available in almost all patients (*n* = 71). A median of 2 units (IQR: 2–4 units) had been transfused per patient. Furthermore, when analysed in more detail, every patient with lrRBCT received a median of 555 ml (IQR: 521–1045 ml) during the perioperative period. The median age of lrRBC units transfused per patient was 17.5 days (IQR: 11.5–27.0 days). Eleven of 71 patients had been transfused with irradiated lrRBC units (15.5%).

### 3.1 Factors associated with administration of lrRBCTs

Factors associated with administration of lrRBC units are summarised in Table 1. Patients receiving perioperative lrRBC units were on average 6.0 years older than patients who did not (*p* = 0.008) and rather had tumours located in the lower limbs (20.6%) than in the trunk (16.3%) or upper limbs (10.3%; *p* = 0.05). Moreover, patients with larger tumours were significantly more likely to receive lrRBC units perioperatively (*p* < 0.001), as were patients with STS situated in the depth (23.3%) or breaching the fascia (19.3%; *p* < 0.001). Also, lrRBC units were more often given in case of liposarcoma as the underlying histological subtype (*p* = 0.022), with the reason most likely being that liposarcomas comprised the histological subtype with the on average largest tumours (10.4 ± 5.3 cm vs. 5.2 ± 3.2 [synovial sarcoma] cm vs. 7.2 ± 5.1 cm [UPS] vs. 7.2 ± 5.2 cm [Others] vs. 8.0 ± 5.4 cm [myxofibrosarcoma]). Need for endoprosthetic (*p* < 0.001) and (neuro‐)vascular reconstruction (*p* = 0.012) were significantly associated with administration of lrRBC units. Gender (*p* = 0.257), margins (*p* = 0.064), amputation (*p* = 0.425), necessity of plastic reconstruction (*p* = 0.721), administration of any CTX (*p* = 0.065), or RTX (*p* = 0.192), were not significantly different between patients receiving and not receiving lrRBC units.

Of those 220 with preoperative laboratory parameters available, those 44 administered lrRBC units perioperatively were far more likely to have low erythrocyte levels (4.2 ± 0.6 T/L vs. 4.7 ± 0.5 T/L; *p* < 0.001), low haemoglobin levels (12.0 ± 2.1 g/dl vs. 14.1 ± 1.6 g/dl; *p* < 0.001), and high CRP‐levels (46.6 ± 67.6 mg/L vs. 11.7 ± 24.3 mg/L; *p* < 0.001) than those 176 patients not requiring lrRBCTs. No significant difference between patients receiving and not receiving lrRBC units regarding preoperative leukocyte levels was found (7.6 ± 2.8 g/L vs. 7.5 ± 2.1 g/L; *p* = 0.831).

Interestingly, differences in CRP levels from pre‐ to postoperative/ ‐transfusion were more marked in patients receiving lrRBCT (*n* = 43), with a mean increase of 45.7 ± 62.3 mg/L in comparison to a mean increase of 26.1 ± 28.3 mg/L for patients without transfusion (*n* = 168; *p* = 0.003). Haemoglobin levels decreased to a greater amount in patients without transfusion from pre‐ to postoperative/−transfusion (−1.9 ± 1.2 T/L vs. 0.1 ± 13.2 T/L; *p* = 0.042).

### 3.2 Prognostic impact of lrRBCTs on LR and DM

In the univariate Fine&Grey model for LR, administration of lrRBC units was not significantly associated with increased risk (SHR: 0.801; 95% CI: 0.364–1.764; *p* = 0.582). Notably, advanced patient age (*p* = 0.024), tumours breaching the fascia (*p* = 0.009, and necessity for plastic reconstruction (*p* = 0.016), were significantly associated with higher LR‐risk (Supplementary Table S1).

In the multivariate model, the only significant factors associated with LR remained tumour location breaching the fascia (*p* = 0.004), advanced patient age (*p* = 0.014), and need for plastic reconstruction (*p* = 0.027), irrespective of lrRBCT (Table 2).

**TABLE 2:** Multivariate Fine&Grey model for LR, with death as competing event (excluding patients having undergone amputation [*n* = 36])

		Multivariate Fine&Grey model for LR (*n* = 381)			
		SHR	95%CI		*p*‐value
			Lower	Upper	
LrRBCT	*No*	1			0.622
	*Yes*	0.811	0.353	1.865	
Age at surgery		1.019	1.004	1.035	**0.014**
Depth	*Superficial*	1			
	*Deep*	0.882	0.445	1.748	0.718
	*Superficial + Deep*	2.846	1.387	5.838	**0.004**
Plastic reconstruction	*No*	1			**0.027**
	*Yes*	1.866	1.073	3.245	

*Note*: *p*‐values in bold highlight significant results.

LrRBCTs were not significantly associated with increased DM‐risk in the univariate Fine&Grey model (SHR: 1.496; 95% CI: 0.948–2.361; *p* = 0.084; Supplementary Table S1). However, advanced patient age (*p* = 0.004), large tumour size (*p* < 0.001), G3 (*p* = 0.011) in comparison to G1 STS, tumours located in the deep (*p* = 0.014) or breaching the fascia (*p* = 0.034) as compared with superficially located STS, and histological subtype “Others” in comparison to myxofibrosarcoma were associated with higher DM‐risk in the univariate Fine&Grey model (Supplementary Table S1). In the multivariate Fine&Grey model, advanced patient age (*p* < 0.001), histological subtypes synovial sarcoma (*p* = 0.021) and “Others” (*p* = 0.001) in comparison to myxofibrosarcoma were independently associated with higher DM‐risk, irrespective of lrRBCT, tumour size, depth, or grading (Table 3). Moreover, liposarcomas (*p* = 0.047) in comparison to myxofibrosarcomas were associated with a lower DM‐risk (Table 3).

**TABLE 3:** Multivariate Fine&Grey model for DM, with death as competing event

		Multivariate Fine&Grey model for DM (*n* = 382)			
		SHR	95%CI		*p*‐value
			Lower	Upper	
LrRBCT	*No*	1			0.058
	*Yes*	1.653	0.984	2.778	
Age at surgery		1.036	1.021	1.052	**<0.001**
Tumour size		1.026	0.986	1.068	0.205
Grading	*G1*	1			
	*G2*	2.631	0.775	8.932	0.121
	*G3*	3.234	0.992	10.541	0.052
Depth	*Superficial*	1			
	*Deep*	1.083	0.670	1.751	0.744
	*Superficial + Deep*	1.792	0.997	3.223	0.051
Histology	*Myxofibrosarcoma*	1			
	*Synovial sarcoma*	3.010	1.183	7.660	**0.021**
	*UPS*	1.538	0.867	2.728	0.141
	*Liposarcoma*	0.429	0.186	0.988	**0.047**
	*Other*	2.254	1.380	3.681	**0.001**

*Note*: *p*‐values in bold highlight significant results.

### 3.3 Prognostic influence of lrRBCTs on OS

**FIGURE 1:**
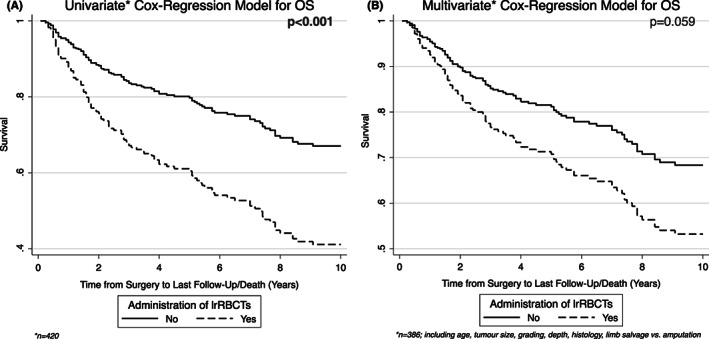
Significant negative impact of lrRBCT transfusion on patient overall survival in the univariate analysis (A), but not in the multivariate analysis (B).

In the univariate Cox‐regression model for OS, lrRBCT (*p* < 0.001; Figure 1A), advanced patient age (*p* < 0.001), large tumour size (*p* < 0.001), amputation (*p* = 0.006), G3 in comparison to G1 STS (*p* = 0.011), and tumours breaching the fascia (*p* = 0.013) as compared with those located superficially were significantly associated with worse outcome (Table 4). Liposarcoma (*p* = 0.028) in comparison to myxofibrosarcoma was associated with improved OS (Table 4).

**TABLE 4:** Univariate and multivariate Cox‐regression models for OS

		Univariate Cox‐regression model for OS				Multivariate Cox‐regression model for OS (*n* = 386)			
		HR	95% CI		*p*‐value	HR	95% CI		*p*‐value
			Lower	Upper			Lower	Upper	
LrRBCT	*No*	1			**<0.001**	1			0.059
	*Yes*	2.222	1.476	3.346		1.658	0.981	2.803	
Age at surgery		1.034	1.020	1.048	**<0.001**	1.033	1.019	1.048	**<0.001**
Gender	*Male*	1			0.389				
	*Female*	0.847	0.580	1.236					
Localisation	*Upper extremity*	1							
	*Lower extremity*	0.939	0.611	1.443	0.775				
	*Trunk*	1.053	0.527	2.192	0.884				
Tumour size		1.058	1.028	1.089	**<0.001**	1.024	0.983	1.067	0.254
Grading	*G1*	1				1			
	*G2*	2.857	0.857	9.517	0.087	2.575	0.757	8.756	0.130
	*G3*	4.443	1.404	14.063	**0.011**	3.245	0.996	10.575	0.051
Depth	*Superficial*	1				1			
	*Deep*	1.167	0.751	1.815	0.492	1.046	0.645	1.696	0.857
	*Superficial + Deep*	2.005	1.156	3.479	**0.013**	1.762	0.979	3.171	0.059
Histology	Myxofibrosarcoma	1				1			
	Synovial sarcoma	0.891	0.396	2.006	0.780	2.332	0.887	6.132	0.086
	UPS	1.551	0.894	2.691	0.119	1.547	0.872	2.744	0.136
	Liposarcoma	0.401	0.178	0.904	**0.028**	0.440	0.191	1.013	0.054
	Other	1.481	0.942	2.328	0.089	2.298	1.413	3.737	**0.001**
Margins	*R0*	1			0.112				
	*R1/2*	1.437	0.919	2.246					
Amputation	*No*	1			**0.006**	1			0.055
	*Yes*	2.251	1.258	4.029		1.860	0.986	3.506	
(Neuro)‐vascular reconstruction	*No*	1			0.415				
	*Yes*	0.727	0.338	1.565					
Plastic reconstruction	*No*	1			0.427				
	*Yes*	0.855	0.581	1.259					
Endoprosthetic reconstruction	*No*	1			0.867				
	*Yes*	0.946	0.493	1.814					
Any CTX	*No*	1			0.276				
	*Yes*	1.303	0.810	2.098					
Any RTX	*No*	1			0.571				
	*Yes*	0.889	0.592	1.335					

*Note*: *p*‐values in bold highlight significant results.

In the multivariate Cox‐regression model, the significant impact of lrRBCT on OS was lost (*p* = 0.059; Figure 1B), whilst advanced patient age (*p* < 0.001) and histological subtype “Others” (*p* = 0.001) in comparison to myxofibrosarcoma were associated with worse OS, irrespective of tumour size, grading, amputation status, and depth (Table 4).

Notably, in a subgroup analysis of those patients with laboratory parameters available prior to surgery, the inclusion of preoperative haemoglobin (HR: 0.809; 95%CI: 0.684–0.57; *p* = 0.014) and CRP‐levels (0.998; 95%CI: 0.684–0.957; *p* = 0.625) – both significantly associated with altered OS in the univariate analysis – further diminished the effect of lrRBCT on OS (HR: 1.677; 95% CI: 0.806–3.492; *p* = 0.167), irrespective of age (*p* = 0.011), amputation status (*p* = 0.023), grading, tumour size, depth, or histological subtype (all *p* > 0.05).

### 3.4 Influence of age and amount of transfused lrRBCTs on OS

As the amount of transfused blood as well as the age of stored lrRBC units after donation may alter patients' overall prognosis, we separately analysed the potential impact of lrRBC units' age, transfused quantity and irradiated versus non‐irradiated lrRBC units on OS.

The amount of lrRBCTs (median of 555 ml) had no significant association on OS (HR: 0.579; 95%CI: 0.285–1.176; *p* = 0.131). Neither there was a significant association of irradiated lrRBC units on patients' OS (HR: 0.770; 95% CI: 0.269–2.206; *p* = 0.627). Furthermore, advanced age of lrRBC units (~17.5 days) was not significantly associated with altered OS (HR: 0.764; 95%CI: 0.377–1.549; *p* = 0.456).

## 4 DISCUSSION

According to the present retrospective single‐centre study, older patients, those with large, high grade (G3) STS of the lower extremities located in the depth or breaching the fascia, undergoing endoprosthetic or (neuro)‐vascular reconstruction, are more likely to require perioperative allogeneic lrRBC units. The association of lrRBCT regarding worse OS in the univariate setting is lost in the multivariate analysis including age, size, grading, depth and histological subtype. Of note, in a further subgroup analysis of patients with preoperative laboratory parameters available, a strong and independent negative impact of low haemoglobin levels on OS is present, whilst the potential impact of lrRBCT is further diminished. In addition, there is no significant impact of lrRBCT on LR or DM. Furthermore, neither the amount of lrRBC units given to patients nor the age of the transfused blood products is significantly associated with altered OS.

Similar to the study by *Heslin* et al., patients in our cohort requiring lrRBCT had significantly larger tumours than patients not undergoing perioperative lrRBCT, and rather had STS located deep to the fascia or breaching it.^15^ Also, patients with G3 STS in comparison to G1 or G2 STS were more likely to be administered lrRBC units, corroborating the results by *Newcomer* et al. in a retrospective study on 99 patients with thigh STS.^9^

In line with the observations by *Rosenberg* et al.^10^ and *Heslin* et al.,^15^ administration of lrRBC units was associated with reduced OS in the univariate analysis. However, whilst *Rosenberg* et al. also reported a significant negative impact of lrRBCT on patients' DM‐free survival, neither in our cohort nor in the one by *Heslin* et al.,^15^ lrRBCT was associated with increased risk for DM. Regarding LR, no significantly altered risk was observed upon administration of lrRBC units, which is in line with reports by *Newcomer* et al.,^9^ and *Heslin* et al.^15^

The lower the haemoglobin levels, the more likely cancer patients require allogeneic lrRBCT. This was likewise observed in the present cohort, with a mean difference in preoperative haemoglobin levels of 2.1 g/dl between patients requiring or not undergoing lrRBCT perioperatively. As in other malignancies,^3^ pre‐treatment anaemia has been associated with poor OS in STS patients,^5^ an observation again confirmed in the present study. Therefore, preoperative optimisation of patients' haemoglobin levels should be strived for, primarily by measures of the hospital‐based patient blood management program (S3 Leitlinie^16^) by intravenous iron substitution and/or ESA, and subsequently in case of persistent need by allogeneic lrRBCT.^1^, ^17^ However, all these treatments involve certain risks that have to be carefully weighed against anticipated benefits. For example, ESA are associated with increased risk for thromboembolic events, regardless of cancer type or initial haemoglobin level.^18^, ^19^, ^20^ LrRBCTs, on the other hand, can lead to specific transfusion reactions, nowadays mainly caused by circulatory overload, alloimmunisation, and accumulation of iron in case of chronic blood transfusions.^6^, ^21^ Furthermore, the aforementioned iron substitution in cancer‐associated anaemia is discussed controversially as the iron could eventually promote tumour cell growth and raise infections risk due to its immune‐modulating role.^22^, ^23^, ^24^, ^25^

As the need for lrRBCT is closely related to negative prognostic parameters including large tumour size and high‐grade disease, it can be explained why the statistical significance of lrRBCT regarding worse OS was lost in the multivariate analysis, similar to the observation by *Heslin* et al.^15^ Yet, a per tendency worse OS in patients receiving lrRBCTs was still observed after adjusting for age, size, grading, depth, histology, and limb salvage surgery versus amputation. This negative association may, on the one hand, be explained by the fact that use of lrRBCT leads to transfusion‐related immunomodulation. Thereby the recipient's immune system is weakened,^1^, ^6^ resulting in worse outcome in STS,^10^ gastric cancer,^26^ colorectal cancer,^27^ lung cancer,^28^ and hepatocellular carcinoma.^29^ In contrast to the results obtained from these observational trials with low scientific evidence, large multicentre randomised clinical trials, meta‐analyses, and international recommendations did not confirm a causal relationship between allogeneic lrRBCT and increased mortality in cancer patients due to TRIM.^30^ Also, the quality of lrRBCTs has most likely improved over the years, with the number of cells other than erythrocytes today reduced to a minimum. Therefore, immune modulating effects exerted by potentially remnant cells may have become less significant. However, also transfused erythrocytes themselves may still contribute to TRIM, with their endothelial adhesion leading to endothelial cell‐activation and thus alteration of the blood coagulation system.^31^, ^32^

On the other hand – and equally important – a potential underlying bias due to the strong association between lrRBCT and low haemoglobin levels, with preoperative anaemia itself being a significant negative prognosticator in cancer patients,^2^, ^3^, ^4^, ^5^ has to be considered.^16^ The latter theory is strengthened by our subgroup analysis of patients with preoperative laboratory parameters available indicating that low haemoglobin levels are a stronger negative predictor for OS than perioperative administration of lrRBC units. Notably, as only two patients in the present cohort were administered lrRBCTs prior to surgery, whilst all others underwent transfusion at the day of surgery or in the postoperative period, no further analysis as to whether timing of lrRBCT had a prognostic impact could be performed. Yet, we discovered a significantly larger increase from pre‐ to postoperative CRP levels in case patients received lrRBCT. An underlying correlation with blood products transfused can only be hypothesised as their impact on elevation in CRP levels – other than inflammatory parameters as interleukin‐6 (not measured in the present study)^33^, ^34^ – is usually rather low.^34^, ^35^ Even more, the extent of surgery – represented by increased blood loss – and subsequent inflammatory response may have likewise resulted in elevated CRP levels.

Although lrRBCT has been linked to transfusion‐related immunomodulation in the past,^36^, ^37^ we did not observe a significant influence of lrRBC units' age on patient OS, being in line with a recent randomised controlled trial reporting no significant impact of the so‐called RBC storage lesion on the clinical outcome (e.g. mortality) of critically ill patients.^38^

Some limitations of the present study have to be mentioned. First and foremost, this study is based on a retrospective evaluation of consecutively treated STS patients at a single centre. Thus, the administration of lrRBCTs was not randomised, which is also evident by factors significantly differing between patients requiring and not requiring lrRBCT. Due to its retrospective nature no statement can be provided regarding the causality of the primary observation that lrRBCTs are associated with lower OS in the univariate analysis. Furthermore, this observation might be also disturbed by a clinically important bias (confounding by indication).^39^ Second, the relatively small number of patients ultimately receiving lrRBCTs in the entire cohort may impede the analysis to which extent age and amount of allogeneic blood products transfused influence prognosis. Third, potential bias due to missing information as incomplete preoperative laboratory parameters owing to the retrospective design of the study has to be considered. Therefore, the herein presented results have to be interpreted bearing these limitations in mind, and warrant further investigation in prospective, preferably randomised clinical trials.

According to the present retrospective study, there is a strong association between lrRBCT and unfavourable prognostic factors as high‐grade STS, advanced patient age, and large tumour size. Thus, unsurprisingly, after accounting for these factors in the multivariate analysis, the negative impact of perioperative lrRBCTs administration on overall survival was lost. Even more, low haemoglobin levels rather than lrRBCT seem to be a strong negative prognosticator. Thus, the detection and adequate treatment of a preoperative anaemia should be strived for to improve patients' prognosis.

## ETHICAL APPROVAL STATEMENT

This study has been approved by the local institutional review board (IRB‐number: 32–225 ex 19/20). Due to the retrospective design of the study, written informed consent was not obtained.

## AUTHOR CONTRIBUTION

Conceptualization – M.P., D.A.B., M.A.S.; methodology – M.A.S., E.M.M., W.H., D.A.B., M.P., A.L.; formal analysis – M.A.S., N.S., E.M.M; data curation – B.L.A., J.S., A.L., D.A.B., W.H.; visualisation – M.A.S., W.H., M.P., D.A.B.; writing original draft – M.A.S., M.P., D.A.B.; writing reviewing & editing – A.L., B.L.A., N.S., J.S., W.H., E.M.M.

## FUNDING INFORMATION

None.

## CONFLICTS OF INTEREST

None of the authors has any conflicts of interest to declare.

## Supporting information


**Table S1** Univariate Fine&Grey models for LR and DM, with death as competing event.

## Data Availability

The original data are available upon reasonable request from the corresponding author.
