# Supplementary material for: Impact of allogeneic red blood cell transfusion on prognosis in soft tissue sarcoma patients. A single‐centre study
Source: Cancer Med. 2022 Jun 28;12(2):1237–46. doi: 10.1002/cam4.4989 (PMC9883560; doi:10.1002/cam4.4989)
Supplement: Supplementary file 1 — Table S1 Univariate Fine&Grey models for LR and DM, with death as competing event. [file CAM4-12-1237-s001.docx]

**Supplementary Material**

**Title:** Impact of allogeneic red blood cell transfusion on prognosis in soft tissue sarcoma patients. A single-centre study.

**Supplementary Table 1.** Univariate Fine&Gray models for LR and DM, with death as competing event.

|  | | **Univariate Fine&Gray Model for LR (n=382)** | | | | **Univariate Fine&Gray Model for DM** | | | |
| --- | --- | --- | --- | --- | --- | --- | --- | --- | --- |
|  |  | **SHR** | **95%CI** | | **p-value** | **SHR** | **95%CI** | | **p-value** |
|  |  |  | **Lower** | **Upper** |  |  | **Lower** | **Upper** |  |
| **LrRBCT** | *No* | 1 |  |  | 0.582 | 1 |  |  | 0.084 |
|  | *Yes* | 0.801 | 0.364 | 1.764 |  | 1.496 | 0.948 | 2.361 |  |
| **Age at Surgery** | | 1.018 | 1.002 | 1.035 | **0.024** | 1.017 | 1.005 | 1.028 | **0.004** |
| **Gender** | *Male* | 1 |  |  | 0.268 | 1 |  |  | 0.567 |
|  | *Female* | 0.732 | 0.422 | 1.270 |  | 1.117 | 0.766 | 1.628 |  |
| **Localisation** | *Upper Extremity* | 1 |  |  |  | 1 |  |  |  |
|  | *Lower Extremity* | 1.173 | 0.614 | 2.240 | 0.630 | 1.547 | 0.961 | 2.492 | 0.073 |
|  | *Trunk* | 1.514 | 0.598 | 3.838 | 0.382 | 1.097 | 0.516 | 2.334 | 0.810 |
| **Tumour Size** | | 1.031 | 0.984 | 1.081 | 0.199 | 1.078 | 1.050 | 1.108 | **<0.001** |
| **Grading** | *G1* | 1 |  |  |  | 1 |  |  |  |
|  | *G2* | 2.505 | 0.571 | 10.977 | 0.223 | 3.166 | 0.986 | 10.162 | 0.053 |
|  | *G3* | 2.872 | 0.696 | 11.843 | 0.145 | 4.299 | 1.403 | 13.169 | **0.011** |
| **Depth** | *Superficial* | 1 |  |  |  | 1 |  |  |  |
|  | *Deep* | 0.730 | 0.386 | 1.380 | 0.333 | 1.796 | 1.127 | 2.861 | **0.014** |
|  | *Superficial+Deep* | 2.541 | 1.267 | 5.095 | **0.009** | 1.975 | 1.054 | 3.704 | **0.034** |
| **Histology** | *Myxofibrosarcoma* | 1 |  |  |  | 1 |  |  |  |
|  | *Synovial sarcoma* | 0.208 | 0.029 | 1.468 | 0.115 | 1.045 | 0.460 | 2.378 | 0.916 |
|  | *UPS* | 0.698 | 0.303 | 1.610 | 0.399 | 1.628 | 0.921 | 2.877 | 0.093 |
|  | *Liposarcoma* | 0.372 | 0.136 | 1.018 | 0.054 | 0.502 | 0.225 | 1.121 | 0.093 |
|  | *Other* | 1.024 | 0.550 | 1.908 | 0.940 | 1.627 | 1.029 | 2.574 | **0.037** |
| **Amputation** | *No* |  | | | | 1 |  |  | 0.675 |
|  | *Yes* |  |  |  |  | 1.162 | 0.575 | 2.348 |  |
| **(Neuro)-vascular reconstruction** | *No* | 1 |  |  | 0.535 | 1 |  |  | 0.737 |
|  | *Yes* | 0.679 | 0.120 | 2.307 |  | 0.876 | 0.403 | 1.900 |  |
| **Plastic Reconstruction** | *No* | 1 |  |  | **0.037** | 1 |  |  | 0.130 |
|  | *Yes* | 1.774 | 1.035 | 3.040 |  | 0.733 | 0.491 | 1.096 |  |
| **Endoprosthetic Reconstruction** | *No* | 1 |  |  | 0.980 | 1 |  |  | 0.299 |
|  | *Yes* | 0.988 | 0.386 | 2.528 |  | 1.369 | 0.756 | 2.476 |  |
| **Margins** | *R0* | 1 |  |  | 0.080 | 1 |  |  | 0.371 |
|  | *R1/2* | 1.706 | 0.938 | 3.102 |  | 1.233 | 0.778 | 1.949 |  |
| **Any CTX** | *No* | 1 |  |  | 0.401 | 1 |  |  | 0.094 |
|  | *Yes* | 1.346 | 0.672 | 2.695 |  | 1.502 | 0.933 | 2.418 |  |
| **Any RTX** | *No* | 1 |  |  | 0.058 | 1 |  |  | 0.776 |
|  | *Yes* | 0.590 | 0.342 | 1.019 |  | 1.060 | 0.708 | 1.589 |  |
| *Univariate Fine&Gray models for LR calculated after excluding patients having undergone amputation (n=36).* | | | | | | | | | |
